# The effect of interactive cognitive-motor training in reducing fall risk in older people: a systematic review

**DOI:** 10.1186/1471-2318-14-107

**Published:** 2014-09-20

**Authors:** Daniel Schoene, Trinidad Valenzuela, Stephen R Lord, Eling D de Bruin

**Affiliations:** Falls and Balance Research Group, Neuroscience Research Australia, Sydney, Australia; School of Public Health and Community Medicine, UNSW, Sydney, Australia; Exercise Science Laboratory, School of Kinesiology, Faculty of Medicine Universidad Finis Terrae, Santiago, Chile; Department of Health Sciences and Technology, Institute of Human Movement Sciences and Sport, ETH Zurich, Wolfgang-Pauli-Str. 27, HIT J 31.2, CH-8093 Zurich, Switzerland; Department of Epidemiology, CAPHRI School for Public Health and Primary Care, Maastricht University, PO Box 616, 6200 MD Maastricht, The Netherlands; Centre for Evidence Based Physiotherapy, Maastricht University, Maastricht, The Netherlands

**Keywords:** Accidental falls, Aged, Interactive cognitive-motor training, Exercise, Balance, Gait, Fear of falling, Cognition, Executive function, Attention

## Abstract

**Background:**

It is well-known physical exercise programs can reduce falls in older people. Recently, several studies have evaluated interactive cognitive-motor training that combines cognitive and gross motor physical exercise components. The aim of this systematic review was to determine the effects of these interactive cognitive-motor interventions on fall risk in older people.

**Methods:**

Studies were identified with searches of the PubMed, EMBASE, and Cochrane CENTRAL databases from their inception up to 31 December 2013. Criteria for inclusion were a) at least one treatment arm that contained an interactive cognitive-motor intervention component; b) a minimum age of 60 or a mean age of 65 years; c) reported falls or at least one physical, psychological or cognitive fall risk factor as an outcome measure; d) published in Dutch, English or German. Single case studies and robot-assisted training interventions were excluded. Due to the diversity of populations included, outcome measures and heterogeneity in study designs, no meta-analyses were conducted.

**Results:**

Thirty-seven studies fulfilled the inclusion criteria. Reporting and methodological quality were often poor and sample sizes were mostly small. One pilot study found balance board training reduced falls and most studies reported training improved physical (e.g. balance and strength) and cognitive (e.g. attention, executive function) measures. Inconsistent results were found for psychological measures related to falls-efficacy. Very few between-group differences were evident when interactive cognitive-motor interventions were compared to traditional training programs.

**Conclusions:**

The review findings provide preliminary evidence that interactive cognitive-motor interventions can improve physical and cognitive fall risk factors in older people, but that the effect of such interventions on falls has not been definitively demonstrated. Interactive cognitive-motor interventions appear to be of equivalent efficacy in ameliorating fall risk as traditional training programs. However, as most studies have methodological limitations, larger, high-quality trials are needed.

**Electronic supplementary material:**

The online version of this article (doi:10.1186/1471-2318-14-107) contains supplementary material, which is available to authorized users.

## Background

Falls are a major public health problem with one in three older people falling at least once a year [1]. Falling is associated with increased mortality [2], injuries [3], loss of independence [4] and adverse psychosocial consequences [5].

Exercise interventions that aim to improve physical risk factors, such as strength and balance training have been shown to reduce fall rates and fall risk [6, 7], fall-related injuries [8] and fear of falling [9, 10] in older people. Systematic review evidence of 44 relevant exercise trials indicates a high exercise dose and challenging balance exercises are important components of successful programs [7]. Presently there is no evidence that cognitive training can lessen fall risk, but there is some evidence suggesting cognitive interventions have a positive impact on cognitive functioning in older populations [11]. The beneficial effects of physical activity decline after exercise cessation [12] and unfortunately low compliance and high drop-out rates in fall prevention studies are often reported [13, 14]. Hence, exercise interventions that facilitate adoption and long term adherence may maximize the efficacy of fall prevention strategies.

Interactive cognitive-motor training (ICMT) requires participants to interact with a computer interface via gross motor movements, such as stepping, receiving immediate visual feedback from the projection screen and include high cost Virtual Reality training as well as less complex and inexpensive exergames [15]. It has been reported that ICMT participation is sufficiently intense to induce exercise-related physiological adaptations in older people [16]. In addition, ICMT requires parallel information processing, selective attention to task-relevant stimuli, inhibition of task-irrelevant stimuli and planning/decision making with respect to the motor execution of the response. These cognitive functions (executive functioning (EF), attention and processing speed) decline with age [17, 18] and if impaired increase fall risk [19]. Importantly, ICMT applications require both cognitive and motor involvement and there is evidence that combined training of cognitive and physical functioning leads to better results than isolated cognitive or physical exercises in older people [20–23].

Because of the potential of ICMT to improve adherence (through the provision of music, direct feedback on performance, positive reinforcement, realistic goal-setting, etc.) and subsequent higher doses of exercise, treatment efficacy may be larger than that achieved with traditionally delivered exercise programs and may lead to sustained improvements. Further, in areas where people have limited access to health care services or where transport is a major barrier for participation, ICMT may provide an effective alternative to enable exercise to be performed at home.

Targeting fall risk factors using ICMT may be effective in reducing falls and improving fall risk factors in older people. Two recent review articles found that exergames are feasible and can improve balance as well as balance confidence in the majority of included studies [24, 25]. However, these reviews were either restricted to commercially available off-the-shelf games, included studies with age groups other than 65 years and over and/or were limited to few risk factors for falls.

Therefore, the current systematic review aimed to 1) synthesize the currently available evidence on the efficacy of ICMT on falls and intrinsic risk factors for falls in older people and 2) determine how such interventions compare to traditionally delivered interventions in reducing the risk of falling in this group.

## Methods

### Literature search strategy

A two-stage process for the identification of potentially relevant studies was used. First electronic databases (Medline (Pubmed), EMBASE (Ovid), Cochrane CENTRAL) were searched from their inception to 31/12/2013. We combined free-text and MeSH terms using a broad range of synonyms, related terms and variant spelling. Second we scanned all reference lists of review articles and included appropriate trials. The Games for Health Journal and the authors own database were hand-searched for relevant articles. No language restrictions were applied to this initial search. Three semantic search loops were used. The first contained terms related to the study design, the second related to ICMT, the third included key words relating to risk factors for falls and fall outcomes. Finally we limited our search to older populations. The search strategy used for PubMed can be found in Additional file 1.

### Inclusion/exclusion criteria

Studies were included if a) at least one treatment arm contained an ICMT component; b) the sample included had a minimum age of 60 years or a mean age of 65; c) at least one physical, psychological or cognitive factor associated with falls or/and fall count data were included as an outcome measure; d) the article was published in Dutch, English or German. In case of multiple publications for one study, all articles were used to obtain maximum information.

Studies were excluded if they were published in abstract form only or designed as a single case study. We also excluded applications in which participants sat while exercising and all robot-based systems, as it was unclear what movements were passive, active or partially supported and therefore different underlying mechanisms may have applied. Finally, studies were excluded if they attempted to change disease-specific outcomes but included if they contained older populations with diseases to investigate fall-related outcomes for which no different underlying mechanisms could be assumed.

Ethical approval was noted for all published papers included in the review. No further ethics approval was sought.

### Data extraction and analysis

Two independent reviewers (DS, EdB) scanned titles and abstracts and full texts if necessary to determine eligibility for each article. Any disagreement was solved by discussion. Extracted data were entered into Microsoft Excel/Word templates specifically developed for this review and piloted using the five first included articles.

The following data were extracted: sample size, population characteristics (age, ethnicity, country, physical function and performance, co-morbidity, falls in previous year), setting (community, hospital, long-term care), ICMT system used, dosage, program of the control group, trial duration, relevant outcomes and assessment instruments, baseline and retest values (between and within group comparisons) and adverse events. Outcome measures of interest included falls as defined by the Prevention of Falls Network Europe [26] and physical, psychological and cognitive measures that have been associated with falls in older people.

Authors were contacted by Email in cases where eligibility could not be established and to clarify any uncertainty regarding intervention content.

### Quality assessment of included studies

Risk of bias was assessed by two independent reviewers (EdB, DS) using the Downs and Black scale for randomized and non-randomized trials [27]. This scale contains 27 items assessing reporting (10 items), internal (13 items) and external (3 items) validity and power (1 item). We modified two items: item 23 (randomisation) and item 27 (study power). For item 23, the method used to generate the randomized sequence (as opposed to a simple statement indicating the trial was randomized) was required to meet this criterion for this item as this is standard in the CONSORT statement. For item 27, authors needed to report if and how they determined their sample size a priori (item 27). Disagreements were solved by discussion or by a third person (TV, SL). For studies where one or more of the authors for this review were involved, the bias risk assessment was undertaken by a third person (TV).

Due to the heterogeneity in study designs, outcome measures and populations used we considered conducting a meta-analysis was not appropriate. A descriptive summary of the results was therefore carried out in lieu. The PRISMA-statement was followed for reporting items of this systematic review [28].

## Results

### Identified studies

**Figure 1:**
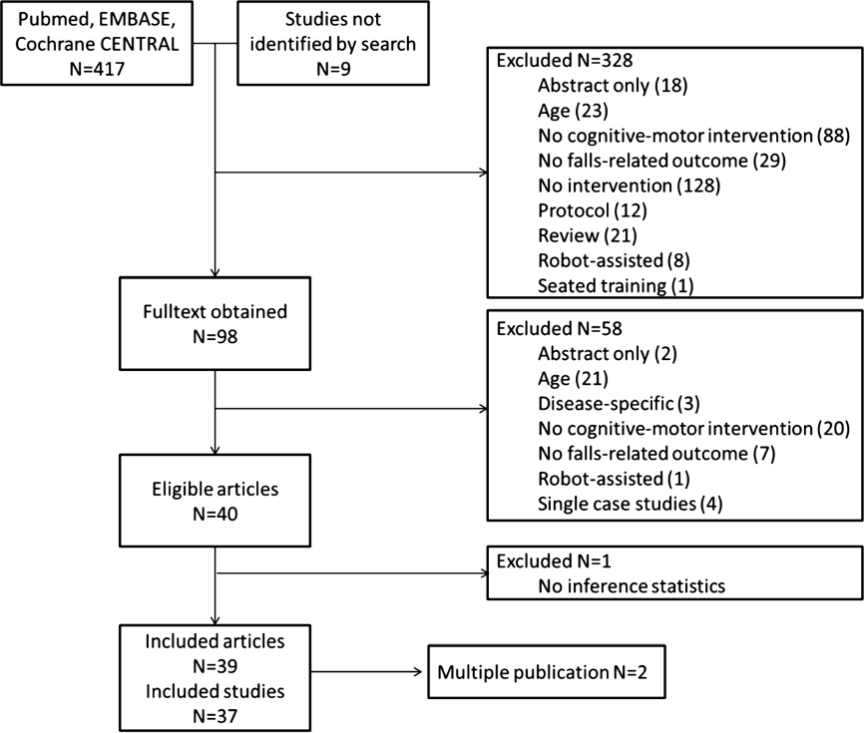
**Flow chart of the study selection process.**

The initial search yielded 426 articles. Of these 98 were obtained as full text and 37 studies were identified as eligible for inclusion in this review - Figure 1 shows the flow chart of the selection process.

### Description of included studies

Tables 1, 2, 3, 4, 5 provide an overview of included studies. Sixteen trials investigated samples with specific medical conditions or functional problems [29–39] including six studies that specifically targeted fallers [40–42] or older people with balance impairments [43–45]. Sixteen studies were conducted in the community [30–32, 38, 44, 46–56], three in independent living facilities [43, 57, 58], six in assisted living facilities [29, 36, 59–62] and one included participants from both the community and aged care facilities [42]. A further five studies were conducted in outpatient clinics [34, 35, 40, 41, 63], two in the in-patient setting [37, 45], and in one case the setting was unclear [33].

**Table 1:** **Step training (dynamic balance, cognitive training)**

Study, sample size	Intervention vs control (content, dose)	Sample characteristics	Main results	
			Within-group	Between-group
**Cognitive-motor only**				
Schoene 2013 [58] N = 32	IG: DDR + CSRT 8 wk, 2-3/wk, 20 min	Independent living (retirement village); age 78 (5), 69–85; able to walk without a walking aid for 20 m, able to step in place unassisted; no disabilities in ADL/IADL functions; no cognitive impairment (MMSE < 24); no neurodegenerative disease; no other health problems affecting stepping ability; no unstable health conditions	*Unpublished*	
			**+**	**+**
			CSRT RT pre 754 (81) post 679 (67) p = .008,	CSRT (F31,1) = 18.203, p < .001,
	CG: Passive			
			CSRT MT pre 252 (44) post 210 (47) p = .035	PPA (F31,1) = 12.706, p < .001,
			PPA pre 1.75 (0.64) post 1.15 (0.85) p < .001	sway velocity (F31,1) = 4.226, p = .049
			Sway mm pre 386 (132) post 301 (133) p = .001	contrast sensitivity (F31,1) = 4.415, p = .044
			proprioception pre 3.0 (1.7) post 2.3 (1.1) p = .091	DT TUG (F31,1) = 4.226, p = .049;
			STS pre 11.5 (2.3) post 10.7 (2.8) p = .032	SST p = .094;
			DT TUG pre 14.1 (5.6) post 11.6 (3.7) p = .002	
			SST pre 50.8 (17.2) post 42.0 (6.8) p = .05	
			**No** hand RT, contrast sensitivity, lower limb strength, AST, TUG, icon-FES, TMT A + B	**No** proprioception, hand RT, lower limb strength, STS, AST, TUG, icon-FES, TMT A + B
Studenski 2010 [55] N= 25	IG: DDR 12 wk, 2/wk, 30 min	Community-dwelling; age 80.2 (5.4), 65+; healthy; able to walk 0.5 miles	**+** narrow walk time pre 5.2 (1.7) change −0.5 (1.6), p = .03 and ABC pre 84.5 (13) change 4.9 (10.1), p = .01;	
	CG: N/A		**No** change DSST **-**balance subscore SPPB	
Lai 2012 [50] N = 30	IG: XMSS 6 wk, 3/wk, 30 min	Community-dwelling; age 72.1 (4.8), 65+; ambulant without walking aids; no neurological disorder; no arthritis or visual or cardiac impairment that affects walking	**+**	
			BBS pre 50.53(4.75) post 53.87(3.56), p = .001,	
			TUG pre 9.54(3.52) post 8.54(2.85), p = .046, sway area eyes open and closed pre (320.80(273.45) post 191.00(70.31), p = .052, pre 342.54(213.67) post 262.20(142.11), p = .092 respectively) sway velocity eyes open and closed pre (9.37(2.30) post 8.10(1.62), p = .046, pre 13.11(5.12) post 11.28(3.55), p = .024 respectively)	
	CG: Passive			
			OLS pre 31.80(18.39) post 48.74(26.67), p = .062	
			MFES pre 131.13(6.56) post 136(6.07), p = .001	
**Cognitive-motor plus other components**				
De Bruin 2011 [59] N = 28	IG: DDR + strength and balance 12 wk, 2/wk, 45-60 min	Assisted living facilities; age 86.2 (7.1), 65+; ambulant without walking aids; no neurological disorder; no arthritis or visual or cardiac impairment that affects walking	**+**	**+**
			DTC: gait speed pre 22 (12.1) post 14.4 (8.6), p = .006, cadence pre 15.8 (13.7) post 10 (7.3), p = .06; stride time pre 20.7 (14.5) post 11.6 (10) p = .004, and step length pre 11.1 (8.3) post 5.5 (5.4) p = .001; FES-I: pre 24.9 (4.5) post 21.9 (5.2), p = .005	DTC: gait speed F(1,26) = 6.25, p = .019, stride time (s) F(1,26) = 5.7, p = .025, step length (cm) F(1,26) = 11.51, p = .002, FES-I: F(1,26) = 2.95, p = .098
	CG: Mostly seated exercises 12 wk, 1/wk, 30-45 min			
			**No**	**No**
			ETGUG, DT step time	DTC Cadence, DTC of step time, ETGUG
Pichierri	IG:	Hostels for the	**+**	**+**
2012a [62] N = 31	DDR + strength and balance 12 wk, 2/wk, 50-60 min	elderly; age 86.2 (4.6), 65+; 50% considered high fall-risk; no major cognitive impairment (MMSE ≥ 22); able to walk 8 m; no acute or chronic unstable illness; adequate vision	ST and DT Improvements throughout most walking conditions;	DT gait speed (U = 26, p = .041, r = .45) and single support time (U = 24, p = .029, r = .48) fast walking condition **No** ST gait, and some parameters DT gait
			DTC decreased throughout most parameters in ST and DT walking **No** FES-I	
				FES-I
	CG: Strength and balance 12 wk, 2/wk, 40 min			
Pichierri 2012b [61] N = 15	IG: DDR + strength and balance 12 wk, 2/wk, 60 min	Care homes; age 84.6 (4), 65+; no major cognitive impairment (MMSE ≥ 22); able to stand upright for 5 min; no acute or chronic unstable illness; adequate vision	**+**	**+**
			step reaction time: time reduction in all assessed temporal parameters ST: −15.7%; DT: −20.1%; step directions with significance and step directions with a trend to significance for step initiation, foot off, and foot contact times for most variables	step reaction time: initiation time of forward steps under DT (U = 9, p = .034, r = .55) and backward steps under DT conditions (U = 10, p = .045, r = .52)
	CG: Non-specific physical activities depending on activity			
				**No**
				ST conditions step reaction time
				DT most other variables of step initiation, lift-off and movement speed

IG intervention group, CG control group, wk week, DDR Dance Dance Revolution, XMSS Xavi measured step system, ADL activities of daily living, IADL instrumental activities of daily living, MMSE Minimental state examination, CSRT choice stepping reaction time, RT reaction time, MT movement time, PPA Physiological Profile Assessment, STS sit-to-stand, TUG timed up & go test, AST alternate step test, icon-FES iconographical falls-efficacy scale, DT dual task, TMT Trailmaking test, SST Stroop Stepping Test, ABC Activities-specific Balance Confidence Scale, DSST Digit Symbol Substitution Test, SPPB Short Physical Performance Battery, BBS Berg Balance Scale, OLS One Leg Stance, MFES Modified Falls Efficacy Scale, DTC dual task costs, FES-I Fall-Efficacy Scale International, ETGUG Extended Timed Get-up-and-go test, ST single task, M-L medio-lateral, A-P anterio-posterior.

**Table 2:** **Balance board training (Standing exercises with feet in place during most exercises, high challenge balance)**

Study, sample size	Intervention vs control (content, dose)	Sample characteristics	Main findings	
			Within-group	Between-group
**Cognitive-motor only**				
Orsega-Smith 2012 [52] N = 34	IG1: WBB balance + strength 4 wk, 2/wk, 30 min	Community-dwelling; age 72.1 (7.8), 55–86; independent, 88% self-reported health good or very good, 0% poor; overweight (mean BMI 27.19 (4.99); high-functioning (ceiling effect in several measures	**+**	**+**
			IG1	IG1 vs CG:
			BBS pre 51.69 (10.05) post 53.13 (8.48), p < .05	BBS mean difference 2.33 (0.77), p = .004
	IG2: WBB balance + strength 8 wk, 2/wk, 30 min			
			STS pre 11.81 (3.62) post 13.69 (3.89), p < .01,	STS mean difference 2.54 (0.69), p = .002
			ADL pre 126.14 (19.53) post 130.36 (12.70), p < .05	IG2 vs CG:
			IG2	BBS p = .05
			BBS pre 54.22 (1.79) post 55.44 (0.89 3), p < .05	STS p = .10
			TUG pre 7.14 (1.08) post 6.74 (0.76), p = .06	
	CG: Passive		ADL pre 130.22 (8.00) post 135.00 (3.50), p < .05	**No**
			ABC pre 87.85 (11.19) post 93.93 (5.52), p < .05	IG1 vs IG2: no sig differences in any measure
				IG1 vs CG: TUG
			**No**	IG2: vs CG:TUG
			IG1: TUG, ABC, FES	
			IG2: STS, FES	
Bieryla 2013 [64] N = 9	IG: WBB balance + strength 3 wk, 3/wk, 30 min	Community-dwelling; age 70+; 70–92; 81.5 (5.5); healthy; able to stand unassisted for 30 minutes; walk a minimum of 6 meters without aid	**+** follow-up (1mo)	
			BBS pre 50 (47.5-51.5) follow-up 53 (52–54), p = .046	
	CG: N/A (reported as RCT but only within group analysis)			
			**No**	
			Post: BBS, FAB, FR , TUG follow-up: FAB, FR, TUG	
Young 2010 [56] N = 6	IG: WBB balance (custom-made) 4 wk, 10 sessions, 20 min	Community-dwelling; age 84.1 (5.1); healthy; no falls past year	**+** sway variability decreased in EC A-P t(5) = 3.042; p = .03,	
			**No**	
	CG: N/A		Sway variability EO and EC M-L	
Kim 2013 [48] N = 32	IG: slow static balance and strength 8 wk, 3/wk, 60 min	Community-dwelling; age IG 68.3 (3.7), CG 66.2 (3.9); 65–75; independently ambulatory; able to stand on 1 leg for 15 seconds without any assistance; no history of orthopedic or neurologic surgery; MMSE ≥ 24; no dementia, cardiovascular disease, headache or dizziness	**+**	**+**
			Hip extension 55%, flexion 29.9%, adduction 48.6%, abduction 41.9%, all p < .001	All hip muscles (p < .001
	CG: passive			
			GRF backward stepping EO 15.4% p = .004, EC 11.5% p = .044	GRF backward stepping test EC p = .028
			GRF cross-over stepping EO 28.7% p < .001, EC 26.6% p < .001	GRF cross-over stepping test EC p = .013
				**No** GRF EO backward and cross-over stepping
Lamoth 2011 [66], Kosse 2011 [49] N = 9	IG: Static balance 6 wk, 3/wk, 20 min	Community-dwelling; age 77 (5), 65+; healthy; highly motivated to exercise; able to walk without aids; no orthopedic or neurological disorders which prevent them from walking without aids or pressing the buttons on the interface; adequate vision; no cognitive impairments		+ BBS p < .01 Figure-of-eight p < .01
	CG: passive (cross-over)			
				**No** Tandem, OLS with EO/EC
Bisson 2007 [46] N = 24	IG: IREX, static standing 10 wk, 2/wk, 30 min	Community-dwelling; age 74.4 (4.3), 65+; no walking aids; no major cognitive impairment (MMSE > 19); no unexplained falls last year; no peripheral neuropathy, an uncontrolled heart problem, severe arthritis, severe back pain, a recent leg injury (last 6 mo), tunnel vision, or any vestibular problem	**+** CB&M pre 58.6, post 64.2, follow-up 64.7 F(2,46) = 14.5, p < 0.01	**No** CB&M, RT , Sway no differences between groups and no training effect
			RT main effect of time F(2,44) = 10.30, p < 0.01, no change between post and follow-up	
			**No** Sway	
	CG: Biofeedback training on force plate 10 wk, 2/wk, 30 min			
Pluchino 2012 [53] N = 27	IG: WBB balance + strength 8 wk, 2/wk, 60 min	Community-dwelling; age 72.5 (8.4) of n = 40; independent; no neurologic disorders affecting balance; no severe cognitive impairment; no major depression; no unstable disease; no severe vestibular problems; no assistance in ADL	**+** DMA score pre 808.75 (98.17) post 761.13 (131.75), p = .036 **No** FROP-COM, TUG, OLS, POMA gait, POMA balance, FR, FES **-** Sway area pre −0.39 (0.23) post 1.65 (1.47), p < .001 (!)	**No** FROP-COM, TUG, OLS, POMA, FR, Sway, dynamic posturography, FES
	CG1: balance			
			Sway velocity pre 1.67 (0.57) post 1.90 (0.71), p = .013	
	CG2: Tai Chi Both 8 wk, 2/wk, 60 min			
Chen 2012 [47] N = 40	IG: Static balance and strength (power) 6 wk, 2/wk, 30 min	Community-dwelling; age 75.9 (7.9), 65+; no dizziness/vertigo, degenerative neurological diseases, stroke, lower limbs fractures, cardio-pulmonary distress and any sensory, visual, auditory or cognitive impairment that would hinder testing procedures; no medication known to affect balance	**+** POMA pre 15.68 (1.38) post 23.33 (2.29), p < .001, +50%	**+** POMA p < .05
				TUG p < .05
	CG: Strength and balance 6 wk, 2/wk, 30 min			STS p < .05
				Power p < .05
				mFES p < .05
			FR pre 16.49 (3.37) post 22.26 (4.21), p < .001, +35%	**No** FR
			TUG pre 17.15 (4.49) post 12.90 (3.07), p = .026, −25%	
			STS pre 17.20 (3.51) post 12.46 (2.99), p = .004, −28%	
			Muscle power pre 4.56 (1.43) post 7.47(2.81), p < .001, +64% mFES pre 5.52 (1.28) post 8.14 (0.94), p = .002, +47%	
Suarez 2006 [41] N = 26	IG: Static balance under changing sensory conditions 6 wk, daily, 40 min	Outpatient clinic; age 73–82; balance disorder; >2 falls in last year; no musculoskeletal disorders, no dementia; no PD or neuropathy	**+** Sway area normal standing pre 10.4 (2.3) post 3.5 (1.4), p < .001	
	CG: N/A			
			Sway area optokinetic stimulation pre 22.4 (4.3) post 10.4 (4.2), p < .001	
			Sway velocity normal standing pre 3.2 (0.5) post 2.4 (0.4), p < .001	
			Sway velocity optokinetic stimulation pre 4.9 (1.4) post 2.9 (0.3), p < .001	
Duque 2013 [40] N = 28 (within) N = 58 (between)	IG: Static balance under changing sensory conditions plus ususal care (sham) 6 wk, 2/wk, 30 min	Community-dwelling; age 65+; IG 79.3 (10); CG 75 (8); falls and fracture clinic; at least 1 fall past 6 mo; poor balance; ambulate independently with a cane or walker; able to stand unaided for 60secs; MMSE ≥ 22; no PD, or neuromuscular condition; GDS ≤ 7; no severe visual impairment	**+** 6 wk	**+** 9mo falls 1.1 (0.7) vs CG 2 (0.2), p < .01
			LOS 31%, p < .01 Sway area EC hard surface −33%; EC foam −52%, optokinetic stimulation 25%, Sway velocity vertical 50%, horizontal 33%, all p < .01	LOS, p < .01 Sway area optokinetic stimulation, p < .01
	CG: Usual care (Sham)			
				Sway velocity horizontal and vertical optokinetic stimulation, p < .01
				SAFFE , p < .01
				**No** Sway area standing hard surface/foam
Padala 2012 [36] N = 22	IG: WBB balance + strength 8 wk, 5/wk, 30 min	Assisted living facility; age 80.5 (7.5), 60+; mild AD; MMSE ≥ 18; excluded: myocardial infarction, transient ischemic attack or stroke in the previous 6 mo, serious mental illness which impacted memory, active cancer diagnosis with the exception of skin cancer, poor prognosis for survival (e.g., severe congestive heart failure), severe sensory (visual or auditory) or musculoskeletal impairments, or a required use of a wheel-chair for ambulation; 44% walking aid; mean 3.2 comorbidities	**+** BBS change 6.27 (5.27), p003	**No** BBS, POMA, TUG, ADL, IADLs, MMSE
	CG: Walking 8 wk, 5/wk, 30 min			
			POMA change 1.82 (2.04), p = .013	
			**No** TUG, ADL, IADL, MMSE	
Szturm 2011 [63] N = 27	IG: static balance on firm or compliant surface 8 wk, 2/wk, 45 min	Geriatric day hospital; age 80.7 (6.5), 65–85; no cognitive impairment (MMSE > 24); independent ambulant; no condition or disability that prevents participation; 89% walking aids; mean gait speed <0.7 m/s	**+** BBS p < .001 TUG p = .07 LOB p = .03 ABC p < .05	**+** BBS t = 5.9, df = 24, p < .001
				TUG t = 1.87, df = 25, p = .08
			**No** Gait speed	
				LOB U = 37.2, p = .007
				ABC U = 44.5, p = .02
				**No**
				Gait speed
	CG: Strength, aerobics, balance			
Yen 2011 [34] N = 42	IG: Static balance with tilt 6 wk, 2/wk, 30 min CG1: balance (incl. tilt board) 6 wk, 2/wk, 30 min	Outpatient clinic; age 70.7 (6.4); idiopathic PD (Hoehn and Yahr stages II and III); no cognitive impairment (MMSE > 24); no uncontrolled chronic diseases; no other neurological, cardiovascular or orthopaedic disorders affecting postural stability; no on-off motor fluctuation; no dyskinesia > grade 3 (UPDRS)	**+** SOT-6 pre 37.4 (25.3-49.4) post 54.3 (44.1-64.5) follow-up 48.6 (36.8-60.4), p < .05/3	**+** Vs CG 2: DT SOT-6, p < .05/3
			DT SOT-6 pre 39.9 (27.9-52.0) post 55.3 (43.7-66.9) follow-up 52.6 (41.3-66.9), p < .05/3	**No** Vs CG 1: no in any measures Vs CG 2: ST SOT-6
	CG2: none			
				ST SOT 1–5 DT SOT 1–5 Verbal RT
			**No** SOT 1–5 Verbal RT DT SOT 1-5	
**Cognitive-motor plus other components**				
Franco 2012 [57] N = 32	IG: WBB plus strength and balance 3 wk, 2/wk, 10-15 min + daily 15 min	Independent-living facility; age 78.3 (6); able to walk independently; adequate vision; able to stand for at least 2 min; no reduced weight-bearing capability; cognitively able to understand instructions	**+** BBS F(1,29) = 17.034, p < .001, change 3.55 (5.03)	**No** BBS, POMA
			POMA F(1,29) = 9.715, p < .004, change 0.91 (2.39)	
	CG1: strength and balance 3 wk, 2/wk, 30-45 min			
	CG2: none			
Fung 2012 [35] N = 50	IG: WBB plus strength and balance (TKR)	Outpatient clinic; age 68 (11); following knee replacement; full lower extremity weight bearing; no active painful OA in lower limb; no visual impairment		**No** knee extension, knee flexion and ABC
	LOS, 2/wk, 15 min + 2/wk, 60 min?			
	CG: Balance + strength			
	LOS, 2/wk, 60 min			
Griffin 2012 [44] N = 65	IG: WBB plus strength and balance 7 wk? CG: strength and balance 7 wk?	Age 83.2 (5.5), 67–90; met the existing criteria to join the falls prevention training group (poor performance TUG, FR, 180 degree turn, flexibility);	**+**TUG −17% FR	**+** TUG FR
			**No** OLS	**No** OLS
Kubicki 2014 [45] N = 32	IG: Fovea, static standing (position/foam/unstable plate according to individual’s ability) + strength and balance; 3 wk, 2/wk, 10 sequences + 3 wk, 3/wk, 30 min	Short-term rehabilitation service; age 71–94; IG 82.2 (6.9), CG 81.5 (5.0); frail (Fried criteria); balance disorder; able to stand unassisted; multiple causes for hospitalisation; no pyramidal or extra-pyramidal syndrome or neuropathy; MMSE ≥24; gait speed = 0.65 (0.23)	**+** Hand RT (ms) pre 605 (244) post 446 (110), p < .05	**+** Hand RT F1,29 = 5.057, p = 0.032
				**No** Sway (mean velocity) TUG ST gait DT gait
				-Sway velocity (APA) F(1,29) = 8.031, p < 0.01 (!)
	CG: strength and balance; 3 wk, 3/wk, 30 min			
				Sway velocity (acc) p = .075

(!) there exists inconsistency in the literature regarding the interpretation of postural sway score changes. Here we assume that an increase in sway is a negative finding.

IG intervention group, CG control group, wk week, WBB wii balance board, MMSE Minimental State Examination, GDS Geriatric Depression Scale, ADL Activities of daily living, AD Alzheimer’s Disease, PD Parkinson’s Disease, UPDRS Unified Parkinson’s Disease Rating Scale, TUG Timed up and go test, FR functional reach test, BBS Berg Balance Scale, STS Sit-to-stand test, ABC Activities-specific Balance Confidence Scale, FES Falls-efficacy Scale, FAB Fullerton Advanced Balance Scale, A-P anterio-posterior, M-L medio-lateral, EO eyes open, EC eyes closed, GRF ground reaction force, CB&M Community Balance and Mobility Scale, RT reaction time, DMA dynamic motion analysis, FROP-Com Falls Risk for Older People–Community Setting, OLS One leg stance test, POMA Performance Oriented Mobility Assessment, MFES modified falls efficacy scale, LOS limits of stability, SAFFE Survey of Activities and Fear of Falling in the Elderly, IADL Instrumental activities of daily living, LOB loss of balance, SOT Sensory Organization Test, DT dual task, ST single task, APA anticipatory postural adjustment, acc acceleration phase.

**Table 3:** **Balance board plus aerobic training (combined balance, strength and aerobics, high challenge balance)**

Study, sample size	Intervention vs Control (content, dose)	Sample characteristics	Main findings	
			Within-group	Between-group
**Cognitive-motor only**				
Agmon 2011 [43] N = 7	IG1: Static balance, strength, aerobics 12 wk, 3/wk, 30 min (5 sessions in first wk)	Continuing care retirement; age 84 (5), 65+; impaired balance (BBS < 52 points); able to walk 4 m without assistive device; no cognitive impairment ty 8(Brief Screen for Cognitive Impairment ≤4); no musculoskeletal or neurologic disorder; no routine use of walking aids; adequate vision and hearing;	**+** BBS pre 49 (2.1) post 53 (1.8), p = .017 Gait speed pre 1.04 (0.2) post 1.33 (0.84) m/s, p = .018	
	CG: N/A			
Maillot 2012 [51] N = 30	IG: Static balance, strength, aerobics 12 wk, 2/wk, 60 min	Community-dwelling; age 73.5 (3.6), 65–78; self-rated health better than bad; sedentary; no visual or auditory impairment; no cognitive impairment (mean MMSE = 29 (1))		**+** Physical Wilk’s Λ = .31, F(10, 18) = 4.06, p = .005
				TUG change −0.94 (0.62) t = 4.53, p < .01
	CG: passive			
				STS change 2.73 (2.28), t = −4.91, p < .01
				EF Wilk’s Λ = .19, F(6, 23) = 15.79, p = .001
				TMT B-A change −15.42 (20.27), t = −2.12, p = .04
				Stroop incongruent (number) change 9.13 (8.80), t = −3.412, p < .01
				Processing speed Wilk’s Λ = .21, F(8, 21) = 9.75, p = .001
				Cancellation (Number) change 10.00 (6.09), t = −5.423, p = .01
				simple RT (ms) change −103 (93), t = 3.962, p < .01
				choice RT (ms) change −104 (74), t = 3.082, p < .01
				**No** Visuo-spatial
Williams 2010 [42] N = 15	IG: Static balance, strength, aerobics 12 wk, 2/wk, individual (most 15 min)	76% community-dwelling; age 76.7 (5.1) of n = 21, 70+; fall past year; no severe cognitive impairment (Abbreviated Mental Test ≥ 7); no wheelchair; 48% walking aid	**+** BBS 4 wk pre 43.7 (9.5) post 48.1 (7.2), p = .02	
			**No** POMA 4/12 wk, FES-I 4/12 wk, BBS 12 wk	
	CG: N/A (reported as CCT but only within group analysis)			
Laver 2012 [37] N = 44	IG: Static balance, strength, aerobics → individual treatment needs	Rehabilitation hospital; age 84.9 (4.5), 65+; no major cognitive impairment (MMSE ≥ 21); able to perform sit to stand without assistance; previously ambulating independently; adequate vision; various causes for hospitalisation	**+** FIM pre 100.45 (16.71) post 108.64 (15.78), p < .001	**+** change in outcome based on number of sessions during hospital stay: IG improved on average 1.26 s/session on the TUG (p = 0.048) and performed better per session on the MBBS (p = 0.042) than CG
			**No** mBBS, TUG, IADL, ABC	
	LOS, 5/wk, 25 min			
				**No** mBBS, TUG, SPPB, IADL , FIM, ABC
	CG: Physio to maximise functional mobility (walking and transfers)			
	LOS, 5/wk, 25 min			
**Cognitive-motor plus other components**				
Mendes 2012 [31] N = 27	IG: Static balance, strength, aerobics + mobility 7 wk, 2/wk, 10 games/2 attempts per game + 30 min	Community-dwelling; age 68.6 (6.4); PD (Hoehn and Yahr I and II); no other problems; no other neurological disorder; no orthopaedic problems; no cognitive impairment (MMSE ≥ 24); GDS (15 items) < 6; no visual or auditory impairment	**+** FR 1 wk p = .003, 3mo p = .02	
	CG: N/A			
Pompeu 2012 [33] N = 32	IG: Static balance, strength, aerobics + strength and mobility 7 wk, 2/wk, 30 min + 30 min	Age 60–85, 67.4 (8.1); idiopathic PD; Hoehn and Yahr stage 1–2; good visual and auditory acuity; no other neurological disorder or orthopaedic disorder; no cognitive impairment (MMSE ≥ 24), no depression (GDS-15 score <6)	**+** BBS pre 52.9 (4.1) post 54.4 (2.2) follow-up54.1 (2.0), p < .005	**No** BBS, OLS, MOCA, DT
			OLS EO pre 23.4 (22.0) post 32.9 (22.6) follow-up 31.2 (23.1), p < .01	
	CG: balance + strength and mobility 7 wk, 2/wk, 30 min + 30 min			
			MOCA pre 20.6 (4.5) post 22.2 (4.5) follow-up 21.8 (4.5), p < .001	
			**No** OLS EC, DT	
Rendon 2012 [54] N = 40	IG: WBB balance + strength plus cycling 6 wk, 3/wk, 35-45 min	Outpatient clinic; community-dwelling; age 60–95, 84.5 (5.3); able to participate in physical activity for 45–60 min; self-reported normal vision; no orthopaedic, neurological or circulatory disorders that prevent participation; 15% walking aids; No participant was able to complete the entire series of exercises without the use of the assistive device at least one time		**+** TUG p = .038
	CG: passive			ABC p = .038
				**No** Depression
Chao 2013 [65] N = 7	IG: Static balance, strength, aerobics + health education and self-efficacy 8 wk, 2/wk, 30 min + 30 min	Assisted living facility; age 80–94; 65+; 86 (5); able to ambulate with or without an assistive device; able to understand instructions; medically stable; no contraindications for exercise; n = 3 cognitive deficit;	**+** BBS pre40.9 (8.5) post 45.1 (6.3), p = .017	
			TUG pre19.4 (5.5) post 15.8 (5.1), p = .063	
	CG: N/A		FES pre31.3 (15.7) post 23.6 (14.1), p = .058	

IG intervention group, CG control group, wk week, LOS length of stay, WBB wii balance board, BBS Berg Balance Scale, MMSE, Minimental State Examination, PD Parkinson’s Disease, GDS Geriatric Depression Scale, TUG Timed up and go test, STS Sit-to-stand test, EF executive function, RT reaction time, POMA Performance Oriented Mobility Assessment, FIM Functional Independence Measure, mBBS modified Berg Balance Scale, IADL Instrumental Activities of Daily living, ABC Activities-specific Balance Confidence Scale, FR Functional Reach test, mo month, OLS One leg stance test, MOCA Montreal Cognitive Assessment, EO eyes open, EC eyes closed, DT dual task, FES Falls-efficacy Scale.

**Table 4:** **Multi-component training (combined aerobic, strength, coordination; low challenge balance)**

Study, sample size	Intervention vs control (content, dose)	Sample characteristics	Main findings	
			Within-group	Between-group
**Cognitive-motor only**				
Lee 2013 [38] N = 55	IG: RT, aerobics, strength, coordination, low level balance (3D, static and dynamic) – higher intensity 10 wk, 2/wk, 50 min, education: twice 50 min	Diabetes; age 65+; IG 73.78 (4.77), CG 74.29 (5.20); independent walking; no intellectual disabilities; 24/55 fall past year	**+** BBS pre 51.67(2.48) post 53.41 (.89), p < .001	
			STS pre 17.51(5.46) post 13.78 (2.86), p < .001	
	CG: N/A (reported as RCT but only within group analysis)		FR pre 28.22 (6.86) post 32.50 (6.31), p < .001	
			TUG pre 11.48 (2.31) post 9.78 (1.58), p < .001	
			OLS pre 15.85 (8.26) 21.75 post (8.11), p < .001	
			Gait speed pre 93.16 (18.97) post 102.87 (16.56), p < .001	
			Cadence pre 101.95 (11.81) post 109.92 (10.94), p < .001	
			mFES pre 6.75 (1.7)9 post 8.11 (1.11), p = .002	
Rosenberg 2010 [30] N = 19	IG: Wii sports unstructured– higher intensity 12 wk, 3/wk, 35 min	Community-dwelling;age 78.7 (8.7); 63–94; subsyndromal depression; no major depression, primary anxiety disorder, bipolar disorder, schizophrenia, or substance use disorder (Mini-International Neuropsychiatric Interview); no cognitive impairment (MMSE < 24); TUG < 14 s; 18% “limited a lot” in performing moderate level physical activity, 35% “limited a little,”, 47% no limitation (SF-36)	**+**Depression (Quick Inventory of Depressive Symptoms) pre 7.8 (3.7) 6 wk 4.8 (2.3), p = .002 post 5.1 (3.0) p = .004	
	CG: N/A			
			Cognition (Repeatable Battery for Assessment of Neurocognitive Status) pre 90.7 (18.0) post 95.3 (16.9), p = .032	
			**No** anxiety (Beck Anxiety Inventory)	
Keogh 2013 [60] N = 26	IG: Wii sports unstructured– higher intensity 8 wk, individual	Residential aged care; age 83 (8); IG 81 (7), CG 85 (7); able to walk at least 10 meters unaided or with a walking aid; sufficient cognitive ability to understand instructions (standard tools such as the MMSE); sedentary		**No** FSST (n = 15/26) p = .199
	CG: passive			
**Cognitive-motor plus other components**				
Hsu 2011 [29] N = 34	IG: Wii sports bowling + strength and balance 4 wk, 2/wk, 20 min + 4 wk, 2-4/wk, ?	Long-term care; age 80, 52–97; self-reported upper extremity dysfunction; no major cognitive impairment (determined by staff); 91% walking aid (including wheelchair)	**No** STS	**No** STS
	CG: strength and balance 4 wk, 2-4/wk, ?			IADL (Nursing Home Physical Performance Test)

IG intervention group, CG control group, wk week, RT reaction time, MMSE Minimental State Examination, TUG Timed up and go test, BBS Berg Balance Scale, STS Sit-to-stand test, FR Functional Reach test, OLS One leg stance, MFES Modified Falls Efficacy Scale, FSST Four Square Step test, ADL Activities of daily living.

**Table 5:** **Aerobic programs (locomotive, low challenge balance)**

Study, sample size	Intervention vs Control (content, dose)	Sample characteristics	Main findings	
			Within-group	Between-group
**Cognitive-motor only**				
Mirelman 2011 [32] N = 20	IG: VR treadmill 6 wk, 3/wk, 45 min	Community-dwelling; age 67.1 (6.5), 55–79; idiopathic PD; moderately impaired (Hoehn and Yahr 2–3); walking difficulties; able to walk unassisted for 5 min; no serious chronic medical condition; no major visual impairment, no major depression; no dementia	**+** gait speed pre 1.16 (0.18) post 1.26 (0.20), p < .05 follow-up 1.28 (0.19), p < .05 Obstacle negotiation	**+** DT gait speed p = .003
	CG: Treadmill (for some outcomes) 6 wk, 3/wk, 45 min			DT stride length p < .001
			- speed pre 0.96 (0.19) post 1.17 (0.22), p < .05cfollow-up 1.17 (0.20), p < .05	
			- stride length pre 148 (17) post 161 (18), p < .05 follow-up 161 (17), p < .05	
			FSST pre 13.3 (2.5) post 11.6 (1.6), p < .05 follow-up 11.9 (1.6), p < .05	
			TMT A pre 69.0 (15.9) post 57.2 (11.9), p = .003	
			TMT B pre 141.4 (34.9) post 120.4 (18.2), P = .05	
			DTC pre 13.9 (14.8) post 6.9 (8.4), p < .05	
			DT gait speed pre 1.01 (0.23) post 1.17 (0.15), p < .05 follow-up 1.13 (0.17), p < .05	
			**No** Gait variability, DTC follow-up	
**Cognitive-motor plus other components**				
Cho 2013 [39] N = 14	IG: VR treadmill + therapeutic exercise (lower extremity muscle strength and gait), occupational therapy, and functional electrical stimulation 6 wk, 3/wk, 30 min + exercise 6 wk, 5/wk, 30 min; OT 6 wk, 5/wk, 30 min; stimulation 6 wk, 5/wk, 20 min	Hemiparesis after stroke within 6mo; stroke rehabilitation ward; age IG 64.57 (4.35), CG 65.14 (4.74); able to walk independently both with and without assistive devices; able to understand and follow simple verbal instructions; MMSE > 24; Brunnstrom score between 1 and 4 for the lower extremity; no serious visual impairment or hearing disorder; no severe heart disease or uncontrolled hypertension and pain; no neurologic or orthopedic disease that might interfere with the study	**+** BBS pre 36.71 (2.28) post 40.85 (1.67), p < .05	**+** BBS p = .011
				TUG p = .013
			TUG pre 22.93 (4.29) post 20.67 (3.73), p < .05	
	CG: Treadmill + therapeutic exercise (lower extremity muscle strength and gait), occupational therapy, and functional electrical stimulation 6 wk, 3/wk, 30 min + exercise 6 wk, 5/wk, 30 min; OT 6 wk, 5/wk, 30 min; stimulation 6 wk, 5/wk, 20 min		Gait speed (cm/s) pre 54.27 (16.18) post 79.67 (13.91), p < .05	Gait speed p = .013
			Cadence pre77.32 (21.91) post 104.04 (10.03), p < .05	
				Cadence p = .035
			step length pre 38.91 (8.24) post 50.51 (9.74), p < .05	
				**No** Spatial gait parameters
			stride length pre 79.21 (16.82) post 99.91 (18.74), p < .05 single limb support pre28.17 (4.77) post 33.64 (2.67), p < .05	

IG intervention group, CG control group, wk week, mo months, VR virtual reality, PD Parkinson’s Disease, MMSE Minimental State Examination, FSST Four Square Step Test, TMT Trailmaking Test, DTC dual task costs, DT dual task, BBS Berg Balance Scale, TUG timed up and go test.

The following ICMT systems were used:

- mats/platforms with pressure sensors [50, 55, 58, 59, 61–63],
- balance boards with pressure sensors: Nintendo Wii balance board (WBB) [31, 33, 35–37, 42–44, 51–54, 56, 57, 64],[65],
- tiltable platforms: SensBalance Fitness board [49]; custom-made [34],
- force plates combined with VR goggles with detection of head movements with/without a foam support surface: (Medicaa Balance Rehabilitation Unit) [40, 41]; uni-axial force plate with four load cells and VR projection on screen [47],
- motion capture systems using cameras: Sony eyetoy [38], Microsoft Kinect [48], GestureTek Interactive Rehabilitation Exercise System [46], using markers placed on shoes while walking on treadmill [32],
- inertial sensors (handheld device): Nintendo Wii [29–31, 33, 37, 42, 43, 51, 60, 65], Fovea [45],
- filmed community walks projected onto a screen [39]

Thirty-four studies delivered the intervention program in one centre-based location [29–42, 44–56, 59–65]. Only two studies administered home-based interventions [43, 58] and in one trial the ICMT component was administered in a centre and complemented by home exercises [57]. Thirty interventions were fully supervised [29, 31–42, 44–47, 49–52, 54–56, 59, 61–65], four were partially supervised [30, 43, 57, 60] and three were unsupervised [48, 53, 58].

The included studies could be classified into five categories according to the physical exercise component of the intervention:

i) Step training - dynamic balance programs involving step training using step pads (pressure sensors); this type of training involved rapid or well-timed steps with weight transfers in multiple directions.
ii) Balance board training - static and dynamic balance programs using balance boards/platforms; this type of training was characterised by feet in place exercises for most movements and therefore only small movements of the centre of mass.
iii) Balance board plus aerobic training - static and dynamic balance plus aerobic training using balance boards and inertial sensors; this type of training involved exercises described under ii) and additional aerobic training (i.e. step aerobics, walking in place).
iv) Multi-component programs with low challenge of balance - full body fitness programs using inertial sensors and/or motion capture devices; this type of training usually simulated sports and involved aerobic, resistance, power and agility components with a low balance challenge.
v) Aerobic programs - locomotive training using VR displays; this type of training included VR treadmill training and involved continuous rhythmic movements with a low balance challenge.

### Methodological quality of included studies

Table 6 summarizes the results of the methodological assessment for the included studies. The quality scores of studies ranged from 5 to 24 points out of a maximum of 28 points. The mean quality score was 16.8 ± 4.5 points, the median value was 17 (IQR 15–19). Some studies investigated “stand-alone” ICMT and reported changes within the training group between baseline and re-assessment only [30, 38, 41–43, 50, 55, 56, 64] while in two studies the ICMT comprised only one component of the training intervention [31, 65]. Other studies compared a “stand alone” ICMT to either passive (or sham) [34, 40, 48, 49, 51, 52, 54, 58],[60] or active [32–34, 36, 37, 46, 47, 53, 63] control activities and some studies added an ICMT as one intervention component to traditional exercises [29, 35, 39, 45, 57, 59, 61, 62]. Studies comparing the ICMT as “stand alone” or as an intervention component to other active forms of exercise did not always use the same dose of exercise prescription. Three studies reported having conducted controlled trials but only reported within-group changes [38, 42, 64].

**Table 6:** **Assessment of methodological quality of included studies using theDowns and Black scale (27)**

First author, year	Risk assesment items																										
	1	2	3	4	5	6	7	8	9	10	11	12	13	14	15	16	17	18	19	20	21	22	23	24	25	26	27
Agmon, 2011 [43]	1	1	1	1	0	1	1	1	1	1	1	0	0	0	0	1	1	1	1	1	1	1	0	0	0	1	0
Bieryla, 2013 [64]	1	1	1	1	0	1	1	0	1	1	0	0	0	0	0	1	1	0	1	1	1	0	0	0	0	1	0
Bisson, 2007 [46]	1	1	1	1	1	0	0	0	0	0	0	0	0	0	0	0	1	1	0	1	1	1	0	0	0	0	0
Chao, 2013 [65]	1	1	1	1	2	1	1	1	1	1	0	0	0	0	0	1	1	1	1	1	1	0	0	0	0	1	0
Chen, 2012 [47]	0	1	1	1	2	1	1	0	1	1	0	0	0	0	0	1	1	0	1	1	1	1	0	0	0	1	1
Cho, 2013 [39]	1	1	1	1	2	1	1	0	1	1	0	0	1	0	1	1	1	1	1	1	1	0	0	1	0	1	0
de Bruin, 2011 [59]	1	1	1	1	2	1	1	1	1	1	0	0	0	0	0	1	1	1	1	1	1	1	0	0	0	0	0
Duque, 2013 [40]	1	1	1	1	2	0	0	0	0	0	1	0	0	0	1	1	1	1	0	1	1	0	0	0	0	1	1
Franco, 2012 [57]	1	1	1	1	2	1	1	0	1	1	1	0	0	0	0	1	1	1	1	1	1	1	0	0	0	0	0
Fung, 2012 [35]	1	1	1	0	2	1	1	0	1	1	1	1	1	0	1	1	1	1	1	1	1	1	1	0	1	1	0
Griffin, 2012 [44]	0	0	0	0	1	1	1	1	0	1	0	0	1	0	0	0	1	0	0	1	1	1	0	0	0	0	0
Hsu, 2011 [29]	1	1	1	1	2	1	1	1	1	1	0	0	0	0	1	1	1	1	1	1	1	1	1	1	0	1	0
Keogh, 2013 [60]	1	1	1	1	2	1	1	1	1	1	0	0	1	0	0	1	1	1	1	1	1	1	0	0	1	0	0
Kim, 2013 [48]	1	1	1	1	1	1	1	0	1	1	0	0	0	0	1	1	1	1	1	1	1	0	0	0	0	1	0
Kosse, 2011 [49]/Lamoth, 2011 [66]	1	1	1	1	0	0	0	0	0	0	0	0	0	0	0	1	1	1	0	1	1	1	0	0	0	0	0
Kubicki, 2014 [45]	1	1	1	0	2	1	1	0	1	1	0	0	0	0	0	1	1	1	0	1	1	0	1	0	0	0	0
Lai, 2012 [50]	1	1	1	0	1	1	1	0	0	1	0	0	0	0	1	1	1	0	1	1	1	1	0	0	0	1	0
Laver, 2012 [37]	1	1	1	1	2	1	1	1	1	1	1	0	1	0	1	1	1	1	1	1	1	1	1	0	1	1	0
Lee, 2013 [38]	1	1	1	1	2	1	1	0	1	1	0	0	0	0	0	1	1	0	1	1	1	0	1	0	0	1	1
Maillot, 2012 [51]	1	1	1	1	1	1	1	0	1	1	0	0	0	1	0	1	1	1	1	1	1	1	1	1	1	1	0
Mendes, 2012 [31]	1	1	1	0	1	1	1	0	0	1	0	0	0	0	0	1	1	0	1	1	0	0	0	0	0	0	1
Mirelman, 2011 [32]	1	1	1	1	0	1	1	1	1	1	0	0	0	0	0	1	1	1	1	1	0	1	0	0	0	1	0
Orsega-Smith, 2012 [52]	1	1	0	1	1	1	1	0	1	0	0	0	0	0	0	1	1	1	1	1	1	1	0	0	0	0	0
Padala, 2012 [36]	1	1	1	1	2	1	1	1	1	1	0	0	0	0	0	1	1	1	1	1	1	0	1	0	0	1	0
Pichierri, 2012a [62]	1	1	1	1	2	1	1	0	1	1	1	0	0	0	0	1	1	1	0	1	1	1	1	0	0	0	0
Pichierri, 2012b [61]	1	1	1	1	2	1	1	1	1	1	1	0	0	0	0	1	1	1	0	1	1	1	1	0	0	0	0
Pluchino, 2012 [53]	1	1	0	1	0	1	1	1	1	1	0	1	1	0	0	1	1	1	0	1	1	0	1	1	0	0	0
Pompeu, 2012 [33]	1	1	1	1	2	1	1	1	1	1	0	0	1	0	1	1	1	1	1	1	0	1	1	1	1	1	1
Rendon, 2012 [54]	1	1	1	1	0	1	0	0	1	1	0	0	0	0	1	1	0	1	1	1	1	0	0	0	0	1	0
Rosenberg, 2010 [30]	1	1	1	1	0	1	1	1	1	1	0	0	0	0	0	1	1	1	1	1	1	1	0	0	0	1	0
Schoene, 2013 [58]	1	1	1	1	2	1	1	1	1	1	0	0	0	0	1	1	1	1	1	1	1	1	1	1	1	1	1
Studenski, 2010 [55]	1	1	1	0	0	1	1	1	1	1	0	0	0	0	0	1	1	1	0	1	1	1	0	0	0	0	0
Suarez, 2006 [41]	0	1	1	0	0	1	1	0	0	1	0	0	0	0	0	1	1	0	0	1	0	1	0	0	0	0	0
Szturm, 2011 [63]	1	1	1	1	0	0	1	0	1	1	0	0	1	0	1	1	1	1	1	1	1	1	1	0	0	0	0
Williams, 2010 [42]	1	1	1	0	1	1	1	1	1	1	0	0	1	0	0	1	1	0	1	1	0	1	0	0	0	0	0
Yen, 2011 [34]	1	1	1	1	2	1	1	1	1	1	0	0	0	0	1	1	1	1	1	1	1	1	1	1	1	1	1
Young, 2010 [56]	1	0	0	1	0	0	0	0	0	0	0	0	0	0	0	0	1	0	0	1	0	1	0	0	0	0	0

Risk assessment items: Items 1–10 Reporting – 1. hypothesis/aim/objectives described?; 2. Main outcomes described?; 3. Participant characteristics described?; 4. Intervention/s described?; 5. distributions of principal confounders in each group described?; 6. Main findings described?; 7. Provision of estimates of random variability in the data for the main outcomes?; 8. Reporting of adverse events?; 9. Characteristics of participants lost to follow-up described?; 10. Actual probability values reported?; items 11–13 External validity – 11. Participants asked to participate representative for population from which they were recruited?; 12. Participants prepared to participate representative for population from which they were recruited?; 13. staff, places, and facilities where the participants were treated representative of the treatment the majority of participants receive?; items 14–20 Internal validity (bias) – 14. Blinding of study participants?; 15. Blinding of outcome assessors?; 16. If any of the results of the study were based on “data dredging”, was this made clear?; 17. In trials and cohort studies, do the analyses adjust for different lengths of follow-up of participants, or in case–control studies, is the time period between the intervention and outcome the same for cases and controls?; 18. Statistical tests appropriate?; 19. Was compliance with intervention/s reliable?; 20. Were the main outcome measures used accurate (valid and reliable)?; items 21–26 Internal validity (confounding) – 21. Were the participants in different intervention groups (trials and cohort studies) or were the cases and controls (case–control studies) recruited from the same population?; 22. Were study subjects in different intervention groups (trials and cohort studies) or were the cases and controls (case–control studies) recruited over the same period of time?; 23. Randomisation, and if yes procedure described?; 24. Allocation concealment?; 25. adequate adjustment for confounding in the analyses from which the main findings were drawn?; 26. Losses of participants to follow-up taken into account?; item 27 power – 27. Power analysis done a priori?; ratings: no = 0, unable to determine = 0, yes = 1; rating item 5: no = 0, partially = 1, yes = 2.

There was poor reporting on randomisation procedures, allocation concealment and blinding. Generally the sample sizes of the included studies were small (range 6–65) with only seven studies conducting sample size analysis a priori, limiting the conclusions that can be drawn; e.g. low power to detect treatment effects. We therefore considered statistical trends (p < .1) as an indication for differences. A multitude of tests, especially for balance were used, with many test measures used in a few studies only. The descriptions of interventions were sometimes inadequate and therefore only partially reproducible.

### Findings for ICMT on risk factors for falls in older people

i) Step training

Six studies with a total of 161 participants investigated the effect of step training interventions (ICMT only [50, 55, 58], ICMT plus other intervention components [59, 61, 62]) (Table 1). No interactive cognitive-motor step training intervention reported results for falls and none were powered to do so. One RCT reported a significant reduction in fall risk as measured with the physiological profile assessment [58].

Exergame step training has also been reported to improve step velocity (reaction time, movement time) [58, 61], step accuracy [50, 62] and measures of static and dynamic balance [50, 55, 58, 62]. Inconsistent results were found for mobility (timed up and go test) [50, 58, 59] and balance confidence and falls-efficacy [50, 55, 58, 59, 62]. Two studies reported step training did not lead to improvements in pen and paper tests of attention and EF [55, 58]. However, several studies have shown step training improves measures of dual tasking [58, 59, 61, 62] and performance in a test that combines stepping and EF [58].

ii) Balance board training

Seventeen studies involving 505 participants have investigated the effect of balance board interventions (ICMT only [34, 36, 40, 41, 46–49, 52, 53, 56],[63, 64], ICMT plus other intervention components [35, 44, 45, 57]) (Table 2). One controlled trial found that a balance training with the feet in place under changing sensory conditions over six weeks significantly reduced falls over a nine month period (IG 1.1 ± 0.7 vs CG 2 ± 0.2, p < .01) [40]. Another study used the FROP-Com to determine fall risk of participants, but found no improvement after eight weeks of training [53].

Consistently, studies have shown balance board training can improve performance in balance batteries (e.g. BBS, POMA) between baseline and re-assessment [36, 46, 47, 52, 57, 63, 64]. Some studies have also reported significant between-group differences using passive [49, 52] and active [47, 63] control groups, whereas others have not - passive control: [57]; active control: [36, 46, 53, 57]. Balance board training has been shown to improve postural sway in the majority of uncontrolled trials after training [40, 41, 56, 66] and when compared to a sham control group [40]. However, balance board training with the IREX Juggler application was found to be ineffective in reducing sway in healthy older people [46], and two studies have reported increases in sway after ICMT [45, 53]. Balance board training has been found to improve strength and power measures after training [47, 48, 52] and when compared to passive [52] and active [47] controls. However, in one study, no between-group difference was found in patients after knee replacement using the Wii balance board as an adjunct to standard rehabilitation [35].

There are inconsistent results for the efficacy of balance board training with respect to falls-efficacy and balance confidence [35, 47, 52, 53, 63]. Few balance board interventions have reported on changes in cognitive performance, including tasks under divided attention. Padala et al. found no improvements in global cognition (MMSE) scores after an eight week training program in people with mild Alzheimer’s disease [36]. In relation to dual task performance, Yen and colleagues found improvements in sway under divided attention when relying more on vestibular feedback [34], but Kubicki et al. found that the use of a platform as an adjunct to standard strength and balance training did not improve dual task gait speed compared to strength and balance training only in frail older people [45].

iii) Balance board plus aerobic training

Eight studies with a combined sample of 202 participants investigated the effect of combined balance board and aerobic training interventions (ICMT only [37, 42, 43, 51], and ICMT plus other intervention components [31, 33, 54, 65]) (Table 3). None of these studies reported results for falls and none were powered to do so.

Combined balance board and aerobic training improved static and dynamic balance [31, 33, 42, 43, 65] and mobility [51, 65] in several studies. However, such training was not effective for these outcomes in a geriatric hospital setting [37] or as an adjunct to mobility training in PD patients [33]. Wii balance board and bicycle training improved depression scores after six weeks training [54], but inconsistent results have been reported for measures of balance confidence and falls-efficacy [37, 42, 65].

Two studies investigated the impact of combined balance board and aerobic training on cognitive measures. In the study by Maillot et al., 12 weeks of Wii training improved EF and processing speed but not visuo-spatial skills in sedentary older people [51]. In the second by Pompeu et al., PD patients improved their global cognitive function (MOCA) after seven weeks of Wii and traditional mobility exercises but no between-group difference was apparent when Wii training was compared to traditional training of a similar dose [33]. This intervention also did not lead to improvements in dual task performance.

iv) Multi-component programs with low challenge of balance

Four studies involving 134 participants investigated the effect of multi-component interventions (ICMT only [30, 38, 60], ICMT plus other intervention component [29]) (Table 4). No intervention reported results for falls and none were powered to do so. A study using the Sony eyetoy in a higher functioning sample of participants with diabetes demonstrated improved functional measures of static and dynamic balance as well as strength [38]. In contrast, two studies in lower functioning residential aged care participants found no improvements in physical outcomes [29, 60]. The aforementioned study in diabetic people also showed improvements after training in falls-efficacy [38], and Rosenberg et al. found 12 weeks Wii sports training program improved depression scores and global cognitive functioning [30].

v) Aerobic programs

Two studies with a combined sample of 34 participants investigated the effect of aerobic interventions involving VR treadmill training (ICMT only [32], and ICMT plus other intervention components [39]) (Table 5). Neither study reported results for falls or fall-related psychological measures, but both showed improvements in balance and mobility [32, 39]. In the study by Mirelman et al., VR treadmill training improved EF and showed larger improvements in dual task gait performance than regular treadmill training in people with Parkinson’s disease [32].

## Discussion

### Effect of interactive cognitive-motor training on falls

The review findings indicate the effect of ICMT on falls is uncertain. Only one of the 37 studies included falls as an outcome measure and due to its modest size (n = 60), this study could be considered to be of a pilot nature for a fall prevention RCT. Encouragingly, the study found a larger reduction of falls in the training group compared to the control group using standing balance training under different sensory conditions [40], as well as improvements in balance and fear of falling; parameters previously reported as mechanisms of effective fall prevention interventions [7, 67].

### The effect of interactive cognitive-motor training on fall risk parameters

The within-group and passive control group comparisons indicate ICMT can improve balance and strength. The majority of studies placed a strong emphasis on balance - the most important component in effective fall prevention exercise interventions [7]. Clinical test batteries (POMA, BBS) in particular, appeared to be sensitive to change and consistently improved. These test batteries provide combined scores for different functional balance tasks which adds power, reduces measurement error and increases the likelihood of finding valid differences [68]. No studies, however, have reported in which sub-tasks participants improved.

Interestingly, two studies found an increase in sway after feet-in-place training [45, 53]. Higher COP velocity and amplitude predict falls [69] which would suggest that the interventions increased fall risk. However, other authors have suggested that an increase in sway after training may relate to improved compensatory strategies [70]. There have also been inconsistent findings regarding intervention effects on one leg stance, functional reach and timed up and go performance. This may be due to the use of off-the-shelves games in many studies. These were not developed to improve clinical outcomes in older people and therefore may lack the task-specificity and/or lack the training principle of progressive overload [71]. The null findings might also be explained by the small sample sizes in many studies and the related low power of detecting significant differences.

It is also possible that psychological consequences of falling can affect quality of life through reduced confidence and activity restriction [72]. Fear of falling and balance confidence improved after training in the review studies that had durations of more than four weeks. However, improvements in falls-efficacy as measured in most trials with versions of the Falls Efficacy Scale (FES, mFES, FES-I, icon-FES), appeared to be not related to the instrument used, the training content or exercise dose. These findings accord with the literature showing that traditional exercise leads to reduced fear of falling in some studies with no clear indication of superiority of one exercise modality [73]. The review findings also indicated ICMT improved depression scores in people both with and without sub-syndromal depression. Depressive symptoms have been consistently associated with falls in older people [74], and exercise is considered an effective strategy for reducing depressive symptoms [75]. However, whether this is due to physiologic, psychological or cognitive factors remains unclear [76].

Cognition, especially EF and attention, are associated with falls in older people [19], and the association between impaired EF and reduced gait speed is one suggested pathway for this association [77]. ICMT improved gait speed and EF in the majority of the review trials, and especially when tasks involving both cognitive and physical components (such as walking under conditions of divided attention) were included; findings were consistent with the literature indicating that cognitive functioning can be enhanced by physical and cognitive exercise [11, 78]. It has been suggested that exercise overcomes age-related overactivity of executive networks in the prefrontal cortex which facilitates motor actions involved in motor planning [79], and that regular physical activity improves efficiency of executive control during more complex tasks involving switching and conflict resolution [80, 81]. Thus, improved coordinated motor performance, especially under real-life multitask conditions, could be a possible mechanism for ICMT reducing fall risk in older people.

### Comparison of interactive cognitive-motor training with traditional training regimens

In studies that compared ICMT to equivalent training programs (similar content, same dose) most comparisons did not show significant differences, suggesting equivalence of training programs. In a few studies, however, ICMT was found to be better than traditional balance and strength or aerobic training in improving physical and cognitive outcomes [32, 34, 37, 39]. These studies were conducted in clinical settings; possibly indicating higher levels of motivation, higher exercise dose and closer supervision. Three of these four studies were also of high methodological quality, so it is possible that other included studies may have failed to demonstrate differences in physical and cognitive outcome measures due to methodological limitations.

The notion of combining cognitive and physical training is based on interrelationships between cognitive and motor functions [82]. Postural control does not simply consist of automated motor tasks but depends on input from higher cortical centres [83], especially from neural networks associated with attention and EF [84]. In addition to good evidence demonstrating cognitive functions improve following exercise interventions [78] there are also preliminary findings suggesting seated cognitive training has beneficial effects on motor functions [85–87]. For example, Verghese and colleagues found eight weeks of seated computer game play training improved gait speed under single and dual task conditions in low-functioning older people; an effect that could not be accounted for by increased levels of physical activity [87].

Using enriched environments in ICMT that require those central processes in addition to motor execution may improve outcomes more than traditional exercise training due to the ecological validity as well as the involvement and interaction of additional modifiable risk factors. In our review however, we were unable to establish consistent differences in functional domains in favour of ICMT. This heterogeneity may have been due, in part, to the low statistical power of many of the included studies. In a related study with a larger sample that did not include standing exercise, VR bike training significantly improved several measures of executive functioning compared with traditional stationary bike training [88]. This VR training effect also exceeded the sum of effects of separate training regimens as reported in the literature, suggesting a synergistic effect [88]. Other studies support this finding in that they report combined physical and cognitive training leads to larger improvements in cognitive, physical and emotional outcomes compared to physical or cognitive training only [20–23].

The feasibility of the lower-cost ICMT exergames and their equivalence with traditional training programs suggest several advantages. ICMT fulfil several criteria to increase adherence and adoption to effective exercise interventions, such as realistic goal-setting, positive reinforcement while exercising, feedback, and the ability to self-monitor one’s performance [89–91]. In addition, due to their easy use and relative low costs they could be deployed in the homes of older people with possible significant cost savings [92]. However, further research is required in this area as only two studies have applied systems within older people’s homes [43, 58] and no studies have conducted cost-effectiveness, cost–utility or cost–benefit analyses of their interventions.

### Limitations of this review

We acknowledge this review has certain limitations. First, it is possible we neglected some trials that were not published in the main databases or referred to by other articles. Second, studies published in languages other than English, German or Dutch were not included. Third, it was not always possible to accurately describe and characterise the included studies due to inadequate reporting. Additional information sought from study authors was obtained for 14 studies [30–32, 34, 35, 37, 40, 42, 45, 54],[56, 58, 62, 63] which assisted in providing more detailed descriptions of the interventions trialled. Finally, due to the heterogeneity in study designs, outcome measures and populations we were unable to conduct a meta-analysis.

## Conclusions

This review shows that the effect of interactive cognitive-motor training on falls remains unclear with only one study including falls as outcome measure. There is evidence from multiple small studies showing that ICMT improved physical and cognitive factors associated with falls in older people but inconsistent findings have been obtained for psychological measures associated with fear of falling. Limited evidence from few studies suggests that ICMT are equivalent to traditional exercise interventions in their effect on fall risk factors.

These review findings have to be regarded with caution due to methodological issues, small sample sizes and poor reporting of the included studies. There is a need for high-quality trials sufficiently powered to show differences in fall rates between groups. In addition, larger trials are required to identify small but meaningful differences between ICMT groups and equivalent traditional training controls. Underlying mechanisms should be explored to determine the interplay between sensorimotor and cognitive functions. Although cost-saving in theory, no studies have investigated cost-effectiveness of their interventions and only a few studies have administered ICMT in the home setting. Future studies therefore should examine these aspects of trial provision.

## Electronic supplementary material

Additional file 1: **Search strategy used in Pubmed.** (DOCX 15 KB)

## References

[1] Blake AJ, Morgan K, Bendall MJ, Dallosso H, Ebrahim SBJ, Arie THD, Fentem PH, Bassey EJ (1988). Falls by elderly people at home: prevalence and associated factors. Age Ageing.

[2] Sylliaas H, Idland G, Sandvik L, Forsen L, Bergland A (2009). Does mortality of the aged increase with the number of falls? Results from a nine-year follow-up study. Eur J Epidemiol.

[3] Tinetti ME, Doucette J, Claus E, Marottoli R (1995). Risk factors for serious injury during falls by older persons in the community. J Am Geriatr Soc.

[4] Donald IP, Bulpitt CJ (1999). The prognosis of falls in elderly people living at home. Age Ageing.

[5] Zijlstra GAR, van Haastregt JCM, van Eijk JTM, van Rossum E, Stalenhoef PA, Kempen GIJM (2007). Prevalence and correlates of fear of falling, and associated avoidance of activity in the general population of community-living older people. Age Ageing.

[6] Gillespie Lesley D, Robertson MC, Gillespie William J, Sherrington C, Gates S, Clemson Lindy M, Lamb Sarah E: **Interventions for preventing falls in older people living in the community.** *Cochrane Database Syst Rev* 2012, (9)**:**Art. No.: CD007146. 10.1002/14651858.CD007146.pub3 PMC8095069 22972103

[7] Sherrington C, Whitney JC, Lord SR, Herbert RD, Cumming RG, Close JCT (2008). Effective exercise for the prevention of falls: a systematic review and meta-analysis. J Am Geriatr Soc.

[8] Robertson MC, Campbell AJ, Gardner MM, Devlin N (2002). Preventing injuries in older people by preventing falls: a meta-analysis of individual-level data. J Am Geriatr Soc.

[9] Rand D, Miller WC, Yiu J, Eng JJ (2011). Interventions for addressing low balance confidence in older adults: a systematic review and meta-analysis. Age Ageing.

[10] Zijlstra GAR, Van Haastregt JCM, Van Rossum E, Van Eijk JTM, Yardley L, Kempen GIJM (2007). Interventions to reduce fear of falling in community-living older people: a systematic review. J Am Geriatr Soc.

[11] Kueider AM, Parisi JM, Gross AL, Rebok GW (2012). Computerized cognitive training with older adults: a systematic review. PLoS One.

[12] Hauer K, Pfisterer M, Schuler M, Bärtsch P, Oster P (2003). Two years later: a prospective long-term follow-up of a training intervention in geriatric patients with a history of severe falls. Arch Phys Med Rehabil.

[13] Nyman SR, Victor CR (2011). Older people’s recruitment, sustained participation, and adherence to falls prevention interventions in institutional settings: a supplement to the Cochrane systematic review. Age Ageing.

[14] Nyman SR, Victor CR (2012). Older people’s participation in and engagement with falls prevention interventions in community settings: an augment to the cochrane systematic review. Age Ageing.

[15] Pichierri G, Wolf P, Murer K, de Bruin E (2011). Cognitive and cognitive-motor interventions affecting physical functioning: A systematic review. BMC Geriatr.

[16] Peng W, Lin JH, Crouse J (2011). Is playing exergames really exercising? A meta-analysis of energy expenditure in active video games. Cyberpsychology, Behavior, and Social Networking.

[17] Salthouse TA (1996). The processing-speed theory of adult age differences in cognition. Psychol Rev.

[18] Hedden T, Gabrieli JD (2004). Insights into the ageing mind: a view from cognitive neuroscience. Nat Rev Neurosci.

[19] Hsu CL, Nagamatsu LS, Davis JC, Liu-Ambrose T (2012). Examining the relationship between specific cognitive processes and falls risk in older adults: a systematic review. Osteoporos Int.

[20] Fabre C, Chamari K, Mucci P, Masse-Biron J, Prefaut C (2002). Improvement of cognitive function by mental and/or individualized aerobic training in healthy elderly subjects. Int J Sports Med.

[21] Oswald W, Gunzelmann T, Rupprecht R, Hagen B (2006). Differential effects of single versus combined cognitive and physical training with older adults: the SimA study in a 5-year perspective. Eur J Ageing.

[22] Theill N, Schumacher V, Adelsberger R, Martin M, Jancke L (2013). Effects of simultaneously performed cognitive and physical training in older adults. BMC Neurosci.

[23] Silsupadol P, Shumway-Cook A, Lugade V, van Donkelaar P, Chou L-S, Mayr U, Woollacott MH (2009). Effects of single-task versus dual-task training on balance performance in older adults: a double-blind, randomized controlled trial. Arch Phys Med Rehabil.

[24] Pietrzak E, Cotea C, Pullman S: **Using commercial video games for falls prevention in older adults: the Way for the future?** *J Geriatr Phys Ther* 9000. Publish Ahead of Print:10.1519/JPT.1510b1013e3182abe1576e 10.1519/JPT.0b013e3182abe76e 24406711

[25] van Diest M, Lamoth C, Stegenga J, Verkerke G, Postema K (2013). Exergaming for balance training of elderly: state of the art and future developments. J of NeuroEngineering and Rehabilitation.

[26] Lamb SE, Jørstad-Stein EC, Hauer K, Becker C, on behalf of the Prevention of Falls Network E, Outcomes Consensus G (2005). Development of a common outcome data Set for fall injury prevention trials: the prevention of falls network Europe consensus. J Am Geriatr Soc.

[27] Downs SH, Black N (1998). The feasibility of creating a checklist for the assessment of the methodological quality both of randomised and non-randomised studies of health care interventions. J Epidemiol Community Health.

[28] Liberati A, Altman DG, Tetzlaff J, Mulrow C, Gotzsche PC, Ioannidis JP, Clarke M, Devereaux PJ, Kleijnen J, Moher D (2009). The PRISMA statement for reporting systematic reviews and meta-analyses of studies that evaluate health care interventions: explanation and elaboration. J Clin Epidemiol.

[29] Hsu JK, Thibodeau R, Wong SJ, Zukiwsky D, Cecile S, Walton DM (2011). A “Wii” bit of fun: the effects of adding Nintendo Wii(®) Bowling to a standard exercise regimen for residents of long-term care with upper extremity dysfunction. Physiother Theor Pract.

[30] Rosenberg D, Depp CA, Vahia IV, Reichstadt J, Palmer BW, Kerr J, Norman G, Jeste DV (2010). Exergames for subsyndromal depression in older adults: a pilot study of a novel intervention. Am J Geriatr Psychiatr.

[31] Mendes FADS, Pompeu JE, Lobo AM, da Silva KG, Oliveira TDP, Zomignani AP, Piemonte MEP (2012). Motor learning, retention and transfer after virtual-reality-based training in Parkinson’s disease - effect of motor and cognitive demands of games: a longitudinal, controlled clinical study. Physiotherapy (United Kingdom).

[32] Mirelman A, Maidan I, Herman T, Deutsch JE, Giladi N, Hausdorff JM (2011). Virtual reality for gait training: can it induce motor learning to enhance complex walking and reduce fall risk in patients with Parkinson’s disease?. J Gerontol.

[33] Pompeu JE, Mendes FADS, Silva KGD, Lobo AM, Oliveira TDP, Zomignani AP, Piemonte MEP (2012). Effect of Nintendo WiiBased motor and cognitive training on activities of daily living in patients with Parkinson’s disease: a randomised clinical trial. Physiotherapy (United Kingdom).

[34] Yen CY, Lin KH, Hu MH, Wu RM, Lu TW, Lin CH (2011). Effects of virtual reality-augmented balance training on sensory organization and attentional demand for postural control in people with Parkinson disease: a randomized controlled trial. Phys Ther.

[35] Fung V, Ho A, Shaffer J, Chung E, Gomez M (2012). Use of Nintendo Wii Fit In the rehabilitation of outpatients following total knee replacement: A preliminary randomised controlled trial. Physiotherapy (United Kingdom).

[36] Padala KP, Padala PR, Malloy TR, Geske JA, Dubbert PM, Dennis RA, Garner KK, Bopp MM, Burke WJ, Sullivan DH (2012). Wii-Fit for improving gait and balance in an assisted living facility: a pilot study. J Aging Res.

[37] Laver K, George S, Ratcliffe J, Quinn S, Whitehead C, Davies O, Crotty M (2012). Use of an interactive video gaming program compared with conventional physiotherapy for hospitalised older adults: a feasibility trial. Disabil Rehabil.

[38] Lee S, Shin S (2013). Effectiveness of virtual reality using video gaming technology in elderly adults with diabetes mellitus. Diabetes Technol Ther.

[39] Cho KH, Lee WH (2013). Virtual walking training program using a real-world video recording for patients with chronic stroke: a pilot study. Am J P M R.

[40] Duque G, Boersma D, Loza-Diaz G, Hassan S, Suarez H, Geisinger D, Suriyaarachchi P, Sharma A, Demontiero O (2013). Effects of balance training using a virtual-reality system in older fallers. Clin Interv Aging.

[41] Suarez H, Suarez A, Lavinsky L (2006). Postural adaptation in elderly patients with instability and risk of falling after balance training using a virtual-reality system. Int Tinnitus J.

[42] Williams M, Soiza R, Jenkinson A, Stewart A (2010). EXercising with Computers in Later Life (EXCELL) - pilot and feasibility study of the acceptability of the Nintendo® WiiFit in community-dwelling fallers. BMC Res Notes.

[43] Agmon M, Perry CK, Phelan E, Demiris G, Nguyen HQ (2011). A pilot study of Wii Fit exergames to improve balance in older adults. J Geriatr Phys Ther (2001).

[44] Griffin M, Shawis T, Impson R, McCormick D, Taylor MJD (2012). Using the Nintendo Wii as an Intervention in a Falls Prevention Group. J Am Geriatr Soc.

[45] Kubicki A, Bonnetblanc F, Petrement G, Mourey F (2014). Motor-prediction improvements after virtual rehabilitation in geriatrics: frail patients reveal different learning curves for movement and postural control. Neurophysiologie Clinique = Clin Neurophysiol.

[46] Bisson E, Contant B, Sveistrup H, Lajoie Y (2007). Functional balance and dual-task reaction times in older adults are improved by virtual reality and biofeedback training. CyberPsychology and Behavior.

[47] Chen PY, Wei SH, Hsieh WL, Cheen JR, Chen LK, Kao CL (2012). Lower limb power rehabilitation (LLPR) using interactive video game for improvement of balance function in older people. Arch Gerontol Geriatr.

[48] Kim J, Son J, Ko N, Yoon B (2013). Unsupervised virtual reality-based exercise program improves hip muscle strength and balance control in older adults: a pilot study. Arch Phys Med Rehabil.

[49] Kosse NM, Caljouw SR, Vuijk PJ, Lamoth CJC (2011). Exergaming: interactive balance training in healthy community-dwelling older adults. J of Cyber Therapy and Rehabilitation.

[50] Lai CH, Peng CW, Chen YL, Huang CP, Hsiao YL, Chen SC (2013). Effects of interactive video-game based system exercise on the balance of the elderly. Gait & Posture.

[51] Maillot P, Perrot A, Hartley A (2012). Effects of interactive physical-activity video-game training on physical and cognitive function in older adults. Psychol Aging.

[52] Orsega-Smith E, Davis J, Slavish K, Gimbutas L (2012). Wii Fit balance intervention in community-dwelling older adults. Game Health J.

[53] Pluchino A, Lee SY, Asfour S, Roos BA, Signorile JF (2012). Pilot study comparing changes in postural control after training using a video game balance board program and 2 standard activity-based balance intervention programs. Arch Phys Med Rehabil.

[54] Rendon AA, Lohman EB, Thorpe D, Johnson EG, Medina E, Bradley B (2012). The effect of virtual reality gaming on dynamic balance in older adults. Age Ageing.

[55] Studenski S, Perera S, Hile E, Keller V, Spadola-Bogard J, Garcia J (2010). Interactive video dance games for healthy older adults. J of Nutrition, Health and Aging.

[56] Young W, Ferguson S, Brault S, Craig C (2011). Assessing and training standing balance in older adults: a novel approach using the ‘Nintendo Wii’ balance board. Gait & Posture.

[57] Franco JR, Jacobs K, Inzerillo C, Kluzik J (2012). The effect of the Nintendo Wii Fit and exercise in improving balance and quality of life in community dwelling elders. Technol Health Care.

[58] Schoene D, Lord SR, Delbaere K, Severino C, Davies TA, Smith ST (2013). A randomized controlled pilot study of home-based step training in older people using videogame technology. PLoS One.

[59] de Bruin ED, Reith A, Dörflinger M, Murer K (2011). Feasibility of strength-balance training extended with computer game dancing in older people; does it affect dual task costs of walking?. J Nov Physiother.

[60] Keogh JW, Power N, Wooller L, Lucas P, Whatman C (2014). Physical and psychosocial function in residential aged care elders: effect of Nintendo Wii sports games. J Aging Phys Act.

[61] Pichierri G, Coppe A, Lorenzetti S, Murer K, de Bruin ED (2012). The effect of a cognitive-motor intervention on voluntary step execution under single and dual task conditions in older adults: a randomized controlled pilot study. Clin Interv Aging.

[62] Pichierri G, Murer K, de Bruin ED (2012). A cognitive-motor intervention using a dance video game to enhance foot placement accuracy and gait under dual task conditions in older adults: a randomized controlled trial. BMC Geriatr.

[63] Szturm T, Betker AL, Moussavi Z, Desai A, Goodman V (2011). Effects of an interactive computer game exercise regimen on balance impairment in frail community-dwelling older adults: a randomized controlled trial. Phys Ther.

[64] Bieryla KA, Dold NM (2013). Feasibility of Wii Fit training to improve clinical measures of balance in older adults. Clin Interv Aging.

[65] Chao YY, Scherer YK, Wu YW, Lucke KT, Montgomery CA (2013). The feasibility of an intervention combining self-efficacy theory and Wii Fit exergames in assisted living residents: a pilot study. Geriatric Nursing (New York, NY).

[66] Lamoth CJ, Caljouw SR, Postema K (2011). Active video gaming to improve balance in the elderly. Stud Health Technol Inform.

[67] Li F, Fisher KJ, Harmer P, McAuley E (2005). Falls self-efficacy as a mediator of fear of falling in an exercise intervention for older adults. J Gerontol Ser B Psychol Sci Soc Sci.

[68] Rushton JPB, Charles J, Pressley M (1983). Behavioral development and construct validity: the principle of aggregation. Psychol Bull.

[69] Piirtola M, Era P (2006). Force platform measurements as predictors of falls among older people - a review. Gerontology.

[70] van Emmerik RE, van Wegen EE (2002). On the functional aspects of variability in postural control. Exerc Sport Sci Rev.

[71] Kenney LW, Wilmore JH, Costill DL, Kenney LW, Wilmore JH, Costill DL (2011). Principles of Exercise Training. Physiology of Sport and Exercise with Web Study Guide.

[72] Jørstad EC, Hauer K, Becker C, Lamb SE, on behalf of the ProFaNE Group (2005). Measuring the psychological outcomes of falling: a systematic review. J Am Geriatr Soc.

[73] Büla CJ, Monod S, Hoskovec C, Rochat S (2011). Interventions aiming at balance confidence improvement in older adults: an updated review. Gerontology.

[74] Kvelde T, McVeigh C, Toson B, Greenaway M, Lord SR, Delbaere K, Close JCT (2013). Depressive symptomatology as a risk factor for falls in older people: systematic review and meta-analysis. J Am Geriatr Soc.

[75] Bridle C, Spanjers K, Patel S, Atherton NM, Lamb SE (2012). Effect of exercise on depression severity in older people: systematic review and meta-analysis of randomised controlled trials. Br J Psychiatry.

[76] Foley LS, Prapavessis H, Osuch EA, De Pace JA, Murphy BA, Podolinsky NJ (2008). An examination of potential mechanisms for exercise as a treatment for depression: a pilot study. Mental Health and Physical Activity.

[77] Kearney FC, Harwood RH, Gladman JRF, Lincoln N, Masud T (2013). The relationship between executive function and falls and gait abnormalities in older adults: a systematic review. Dement Geriatr Cogn Disord.

[78] Gregory MA, Gill DP, Petrella RJ (2013). Brain health and exercise in older adults. Curr Sports Med Rep.

[79] Berchicci M, Lucci G, Di Russo F (2013). Benefits of physical exercise on the aging brain: the role of the prefrontal cortex. J Gerontol A: Biol Med Sci.

[80] Kamijo K, Takeda Y (2010). Regular physical activity improves executive function during task switching in young adults. Int J Psychophysiol.

[81] Colcombe SJ, Kramer AF, Erickson KI, Scalf P, McAuley E, Cohen NJ, Webb A, Jerome GJ, Marquez DX, Elavsky S (2004). Cardiovascular fitness, cortical plasticity, and aging. Proc Natl Acad Sci U S A.

[82] Schäfer S, Huxhold O, Lindenberger U (2006). Healthy mind in healthy body? A review of sensorimotor–cognitive interdependencies in old age. Eur Rev AgingPhys Act.

[83] Hausdorff J, Yogev G, Springer S, Simon E, Giladi N (2005). Walking is more like catching than tapping: gait in the elderly as a complex cognitive task. Exp Brain Res.

[84] Yogev-Seligmann G, Hausdorff JM, Giladi N (2008). The role of executive function and attention in gait. Mov Disord.

[85] Milman U, Atias H, Weiss A, Mirelman A, Hausdorff JM (2014). Can cognitive remediation improve mobility in patients with Parkinson’s disease? findings from a 12 week pilot study. J of Parkinson’s Disease.

[86] Smith-Ray RL, Hughes SL, Prohaska TR, Little DM, Jurivich DA, Hedeker D (2013). Impact of cognitive training on balance and gait in older adults. J Gerontol Ser B Psychol Sci Soc Sci.

[87] Verghese J, Mahoney J, Ambrose AF, Wang C, Holtzer R (2010). Effect of cognitive remediation on gait in sedentary seniors. J Gerontol A: Biol Med Sci.

[88] Anderson-Hanley C, Arciero PJ, Brickman AM, Nimon JP, Okuma N, Westen SC, Merz ME, Pence BD, Woods JA, Kramer AF, Zimmerman EA (2012). Exergaming and older adult cognition: a cluster randomized clinical trial. Am J Prev Med.

[89] Annesi JJ (2002). Goal-setting protocol in adherence to exercise by Italian adults. Percept Mot Skills.

[90] Noland MP (1989). The effects of self-monitoring and reinforcement on exercise adherence. Res Q Exerc Sport.

[91] Shakudo M, Takegami M, Shibata A, Kuzumaki M, Higashi T, Hayashino Y, Suzukamo Y, Morita S, Katsuki M, Fukuhara S (2011). Effect of feedback in promoting adherence to an exercise programme: a randomized controlled trial. J Eval Clin Pract.

[92] Davis JC, Robertson MC, Ashe MC, Liu-Ambrose T, Khan KM, Marra CA (2010). Does a home-based strength and balance programme in people aged ≥80 years provide the best value for money to prevent falls? A systematic review of economic evaluations of falls prevention interventions. Br J Sports Med.

[CR93] The pre-publication history for this paper can be accessed here:http://www.biomedcentral.com/1471-2318/14/107/prepub

