# Supplementary material for: The effect of interactive cognitive-motor training in reducing fall risk in older people: a systematic review
Source: BMC Geriatr. 2014 Sep 20;14:107. doi: 10.1186/1471-2318-14-107 (PMC4181419; doi:10.1186/1471-2318-14-107)
Supplement: Supplementary file 1 — Additional file 1: Search strategy used in Pubmed. (DOCX 15 KB) [file 12877_2014_1040_MOESM1_ESM.docx]

**Additional file 1: search strategy used in Pubmed**

Search strategy:

(intervention or program or programme or "clinical trial" or "clinical study" or controlled or comparison)

AND

(exergame or exergames or exertainment or "virtual reality" or "virtual environment" or "serious game" or "serious games" or "exertion game" or "exertion games" or "video game" or "video games" or "computer game" or "computer games" or "active games")

AND

(exercise or "exercise therapy" or "accidental falls" or fall-risk or falls-risk or "falls risk" or "fall-risk" or falls or faller or fallers or "physical function" or "functional performance" or fitness or "postural stability" or "activities of daily living" balance or frail or frailty or "functional limitation" or gait or impairment or mobility or "performance test" or "physical activities" or "physical activity" or "physical health" or "physical inactivity" or "physical performance" or power or proprioception or "reaction time" or "response time" or sway or walking or mental or cognit* or “executive function” or “EF” or processing or attention or “dual task” or dual-task or “fear of falling” or “falls efficacy” or “concerns about falling”)
